# Influence of Physical Activity Intervention on Circulating Soluble Receptor for Advanced Glycation end Products in Elderly Subjects

**DOI:** 10.4021/jocmr704w

**Published:** 2011-09-26

**Authors:** Kazuhiko Kotani, Russell Caccavello, Naoki Sakane, Toshiyuki Yamada, Nobuyuki Taniguchi, Alejandro Gugliucci

**Affiliations:** a Department of Clinical Laboratory Medicine, Jichi Medical University, Tochigi, Japan; b Glycation, Oxidation and Disease Laboratory, Touro University-California, CA, USA; c Division of Preventive Medicine, Clinical Research Institute, National Hospital Organization Kyoto Medical Center, Kyoto, Japan

## Abstract

**Background:**

Inflammation, often accompanied by oxidation, caused by advanced glycation end products (AGEs) may be quenched by the soluble receptor for AGEs (sRAGE). The present study aimed to investigate the influence of physical activity on circulating sRAGE, and the association between changes of circulating sRAGE and paraoxonase1 (PON1) activity (as an antioxidative enzyme) in a physical activity intervention study on an elderly subject cohort.

**Methods:**

Serum sRAGE, PON1 activity and cardiometabolic variables were measured in 30 community-dwelling asymptomatic Japanese volunteers (15 men/15 women, mean age 65 years) in the pre- and post-phase of a 6-month interventional program designed to increase physical activity.

**Results:**

The body mass index and sRAGE levels (1103 ± 496 to 1030 ± 437 ng/L, P < 0.05) were significantly reduced during the intervention period. In addition, the change of sRAGE was significantly and inversely correlated with that of PON1 activity, independent of the other cardiometabolic variables (β = - 0.511, P < 0.01).

**Conclusions:**

This study showed a reduction of sRAGE levels, and an inverse correlation between sRAGE and PON1 activity, after the intervention study increasing physical activity on an elderly population. These findings represent a modest but significant modulation of sRAGE by this type of exercise intervention, which warranted future studies on the clinical relevance of sRAGE changes in physical activity.

**Keywords:**

AGEs; RAGE; Paraoxonase1; Exercise; Atherosclerosis

## Introduction

Advanced glycation end products (AGEs), a heterogeneous group of adducts formed on proteins, lipids and nucleotides and by reactive carbonyls, are generated under conditions of hyperglycemia and oxidative stress or a combination of both (e.g., glucose intolerance, renal failure and chronic inflammatory disorders) [1,2]. The sustained high levels of AGEs activates the expression of the receptor for AGEs (RAGE), which is a multiligand member of the immunoglobulin superfamily of cell surface molecules [3-5]. The engagement of RAGE with AGEs (as one class of RAGE ligands) has been implicated in the development of various disorders including vascular disease [2-5]. RAGE has a C-truncated secretory isoform of the receptor protein, termed soluble RAGE (sRAGE), and endogenous sRAGE has recently been identified in humans [2-5]. Soluble RAGE is postulated to play an antagonistic role by competing with the cell surface receptor, thus preventing the adverse effects of RAGE-signaling (namely, nuclear factor-kappa B-mediated RAGE-elicited damage) [2-5]. Indeed, one study reported an inverse relationship between circulating sRAGE levels and cardiometabolic markers in the general population, suggesting that sRAGE levels, as a decoy of AGE, may increase and serve as protective players in common lifestyle-related diseases [6]. On the other hand, another study reported a positive correlation between circulating sRAGE levels and microvascular dysfunction and diabetic nephropathy, suggesting sRAGE may increase as a marker reflective of a proinflammatory state [7]. Thus, many remains to be explored to ascertain the physiological function of endogenous sRAGE, and there is a paucity of information on the regulation of sRAGE levels.

While increases in physical activity and exercise are widely purported to maintain health and minimize or prevent several diseases, functional loss as well as disability, and are essential to healthy ageing in older people, the underlying mechanisms have been incompletely elucidated [8-10]. Although only one study on exercise has shown a non-significant change of circulating sRAGE levels in both coronary artery disease patients and healthy subjects, this was based on a specific study setting of a single bout of bicycle-stressed exercise test [11]. As for the beneficial health effects of physical activity, the antioxidant function of high-density lipoprotein (HDL) has received considerable attention [8-10]. While paraoxonase1 (PON1) is a key antioxidative enzyme in HDL, we have previously shown and others have confirmed that circulating AGEs can reduce PON1 activity [12,13]. However, there are no reports on the association between sRAGE and PON1. Keeping this background in mind, the present intervention study in a cohort of healthy elderly subjects had the following aims: (1) to investigate the influence of physical activity increase on circulating sRAGE, and (2) to observe the association between changes of circulating sRAGE and PON1 activity during an intervention period of increased physical activity.

## Subjects and Methods

A total of 30 community-dwelling Japanese elderly volunteers (15 men/15 women; mean age = 65 ± 3 years) were recruited into a 6-month interventional program. All participants were in good health, not current smokers, not on any medication and had not been diagnosed to have diabetes mellitus nor cardiovascular, liver, kidney, nutritional or collagen diseases. The participants were sedentary (no habits of structured exercise < 30 minutes, twice a week). The present program, which focused on a mild-to-moderate increase in physical activity, included monthly explanatory and motivational sessions to increase physical activity such as daily walking and to instruct on the appropriate methods. In particular, the participants were educated on how to measure their heart rates during a walking exercise in their radial arterial pulses, and recommended to perform the walking at 60% of the maximal heart rate of each individual and at least ≥ 30 minutes. The study was approved by the Jichi Medical University ethics committee, and each subject provided informed consent.

In the pre- and post-phase of this intervention, cardiometabolic variables were measured during an overnight fasted state in all subjects. Body mass index (BMI) was calculated as the weight divided by the square of the height measured in light indoor clothing without shoes. Systolic blood pressure (SBP) and diastolic blood pressure (DBP) was determined in the right arm of each subject in the seated position after a 5-minute rest, using the sphygmomanometer. Serum low-density lipoprotein cholesterol and HDL cholesterol (HDL-C) were determined using homogeneous methods, and serum triglycerides (TG) and plasma glucose were measured using enzymatic methods. Plasma insulin was measured using an enzyme immunoassay (TOSOH Co. Ltd., Tokyo, Japan), and the homeostasis model assessment of insulin resistance (HOMA-IR) was calculated using a formula based on the plasma glucose and insulin levels, as described previously [14].

Serum PON1 activity was kinetically measured using paraoxone as a substrate, from the initial velocity of p-nitrophenol production at 37 °C, and recorded at 405 nm in a Versamax Microplate Reader (Molecular Diagnostics, CA, USA), as described previously [15,16]. Serum sRAGEs were measured by an enzyme-linked immunosorbent assay using the Quantikine Human RAGE Immunoassay (R&D Systems Inc., Minneapolis, MN, USA). It contains NS0-expressed recombinant human RAGE/Fc Chimera and has been shown to accurately quantitate the recombinant factor. Plates were read with a Versamax Microplate Reader (Molecular Diagnostics, CA, USA).

Data are shown as the mean ± standard deviation or the median plus interquartile ranges. Paired t test was used to compare the pre- and post-phase values of respective measured variables. Single linear regression analysis and stepwise multiple linear regression analysis adjusted for measured variables were used to evaluate the correlations between the variables in the pre-phase as well as between the changes between the pre- and post-phase. The magnitude of the changes was calculated by subtracting the pre-phase values from the post-phase values in each case. The variables of TG, insulin and HOMA-IR were log-transformed in these analyses because of their skewed distribution. Due to a close co-linearity of insulin and HOMA-IR (correlation coefficient > 0.8), insulin (instead of HOMA-IR) was entered into the stepwise multivariate model. A P value < 0.05 was considered to be significant.

## Results

Clinical characteristics of the studied subjects are listed in Table 1. There was a modest but significant reduction in BMI levels, and similarly, circulating sRAGE levels were significantly reduced through the present intervention on physical activity. Small changes were observed in the other cardiometabolic variables including PON1 activity.

**Table 1:** Clinical Characteristics in the Pre- to Post-phase of Intervention

**Variable**	**Pre-phase**	**Post-phase**	**P value**
Body mass index, kg/m²	23.6 ± 2.4	23.4 ± 2.5	0.018*
Systolic blood pressure, mmHg	137.1 ± 17.1	136.9 ± 21.1	0.948
Diastolic blood pressure, mmHg	78.6 ± 11.5	79.8 ± 11.5	0.336
LDL-cholesterol, mmol/L	3.47 ± 0.71	3.28 ± 0.70	0.072
Triglycerides, mmol/L	1.19 (0.80-1.43)	1.14 (0.83-1.47)	0.502
HDL-cholesterol, mmol/L	1.83 ± 0.39	1.83 ± 0.39	0.883
Fasting plasma glucose, mmol/L	5.33 ± 0.53	5.25 ± 0.58	0.386
Insulin, µU/mL	4.5 (3.7-6.8)	5.0 (3.7-6.2)	0.749
HOMA-IR	1.15 (0.80-1.62)	1.20 (0.88-1.40)	0.626
PON1, U/L	161.3 ± 49.5	160.6 ± 50.0	0.870
Soluble RAGE, ng/L	1103 ± 496	1030 ± 437	0.019*

LDL: low-density lipoprotein; HDL: high-density lipoprotein; HOMA-IR: homeostasis model assessment of insulin resistance; PON1: paraoxonase1; RAGE: the receptor for advanced glycation end products. Data are expressed as the means ± standard deviations or the medians (interquartile ranges). Significance level (difference between the pre- and post-phase data by a paired t-test [triglycerides, insulin and HOMA-IR were log-transformed]): *P < 0.05.

Univariate correlations between sRAGE and the other cardiometabolic variables are listed in Table 2. In the pre-phase, sRAGE levels were significantly and inversely correlated with BMI and HOMA-IR, respectively. Subsequently, a stepwise multivariate analysis confirmed BMI as the only independent, significant and inverse variable for sRAGE (β = - 0.512, P = 0.004). Furthermore, in terms of the level changes between pre- and post-phase, the change of sRAGE levels was significantly and inversely correlated with that of PON1 activity. A subsequent stepwise multivariate analysis confirmed the change of PON1 as the only independent, significant and inverse variable for that of sRAGE (β = - 0.511, P = 0.004).

**Table 2:** Correlations of Soluble RAGE With Other Variables

**Variable**	**Pre-phase**	**Pre- and post-change**
	**r (P value)**	**r (P value)**
Age, years	0.068 (0.722)	0.188 (0.318)
Gender, male	-0.063 (0.742)	0.011 (0.955)
Body mass index, kg/m²	-0.512 (0.004)**	-0.191 (0.313)
Systolic blood pressure, mmHg	0.048 (0.800)	0.135 (0.476)
Diastolic blood pressure, mmHg	-0.071 (0.710)	0.174 (0.358)
LDL-cholesterol, mmol/L	-0.080 (0.673)	-0.230 (0.221)
Triglycerides, mmol/L	-0.050 (0.792)	0.123 (0.516)
HDL-cholesterol, mmol/L	-0.154 (0.418)	-0.189 (0.317)
Fasting plasma glucose, mmol/L	-0.021 (0.912)	-0.030 (0.874)
Insulin, µU/mL	-0.290 (0.120)	0.041 (0.829)
HOMA-IR	-0.365 (0.047)*	-0.013 (0.944)
PON1, UL	-0.166 (0.381)	-0.511 (0.004)**

RAGE: the receptor for advanced glycation end product; LDL: low-density lipoprotein; HDL: high-density lipoprotein; HOMA-IR: homeostasis model assessment of insulin resistance; PON1: paraoxonase1; Pre-phase: correlation between the pre-phase data in respective variables; Pre- and post-change: correlation between the level changes (the post-phase data minus the pre-phase data) in respective variables (age and gender used the pre-phase data were); r: simple linear regression coefficient between soluble RAGE and another variable; β: multiple linear regression coefficient in a stepwise multivariate analysis for soluble RAGE after controlling for all the listed variables (except for HOMA-IR). Triglycerides, insulin and HOMA-IR were log-transformed for the analyses. Significance level: *P < 0.05; **P < 0.01.

## Discussion

The present study on asymptomatic elderly people showed two new findings: (1) circulating sRAGE levels were significantly reduced during an interventional period of physical activity increase, and (2) an independent, significant and inverse correlation between changes of circulating sRAGE and PON1 activity was observed during the intervention. Because the reduction of sRAGE concentrations was significant but modest, and the role of endogenous sRAGE, in comparison to that in experimental settings, is under scrutiny [7], the clinical relevance of the present study should be confirmed in future studies. However, the present findings showing a factor that modulates sRAGE concentrations are interesting, since sRAGE is considered an important biomarker of cardiometabolic pathophysiology and a possible therapeutic target on various disorders [2-5].

Contrasting our results on the change of sRAGE levels after the interventions, an earlier study has reported a non-significant (with a trend to increase) change of sRAGE levels after a single bout of bicycle-stress exercise [11]. Large differences in the intervention methods and durations, as well as studied populations, make a simple comparison between the earlier study and ours difficult. In addition, our intervention was at a mild-to-moderate increase in physical activity that did not produce apparent changes in blood pressure, glucose or lipid panels; thus, these general cardiometabolic factors did not appear to be involved in the mechanisms of the observed reduction of sRAGE in the present population. However, some previous work on lifestyle modification may help to explain, in part, the potential mechanisms for the reduction of sRAGE. Although the present study did not measure AGEs, we have previously shown that a caloric restriction intervention significantly reduces AGEs [16] and a 3-month physical activity intervention, like ours, reportedly induced a significant reduction of AGEs [17,18]. The reduction of circulating AGEs can inactivate the expression of RAGE, thereby leading to the reduction of sRAGE as a decoy of AGEs [2-5]. This may be an adaptive response to increased physical activity in relatively healthy individuals. As another mechanistic explanation, the improvement of the sedentary status can attenuate inflammation, thereby leading to the reduction of sRAGE as a marker reflective of chronic inflammation [7]. Finally, there may be an increase of the clearance of sRAGE from the circulation as a result of hemodynamic changes induced by the increase in physical activity [3,19].

Regarding the association between sRAGE and cardiometabolic variables including PON1 activity, an independent, significant and inverse correlation between sRAGE and BMI was observed in the pre-phase of the present study. This result is in agreement with earlier findings, suggesting that sRAGE may be protective of cardiometabolic outcomes [6]. A novel result of our study is a clear inverse correlation between changes of sRAGE and PON1 activity, even though the changes of PON1 activity were small during the intervention. Of interest, we have shown and others have confirmed in a recent study that AGEs can reduce PON1 activity [12,13]. Given the prior findings on AGEs [17,18], a reduction of AGEs through physical activity (which can lead to an adaptive reduction of sRAGE as aforementioned) may restore PON1 activity. Despite an inverse but non-significant correlation between sRAGE and PON1 in the pre-phase, the fact that a significant correlation between changes of sRAGE and PON1 was observed during the intervention might also imply an individual difference in the response to physical activity increase, for instance, based on genetic components [20,21]. RAGE binds to several ligand molecules, such as S100-calcium binding protein/calgranulins, high-mobility group protein 1, amyloid-β-peptides and the family of β-sheet fibrils, besides AGEs [2-4], so more studies including these molecules are also warranted.

There are some limitations to our study. The sample size was relatively small. The intervention period was restricted to 6-month. The levels of sRAGE and PON1 activity can be affected by genetic and ethnic factors [19,20]. Future studies with larger and ethnically diverse populations and/or longer follow-up periods are necessary to expand the generizability of our results.

In summary, the present study found a significant reduction of circulating sRAGE levels, and an independent, significant and inverse correlation between changes of circulating sRAGE and PON1 activity after a 6-month intervention to increase physical activity in asymptomatic elderly people. Further research is needed to confirm the present findings and explore the biological mechanisms of this association.
